# Machine Learning-Inspired Hybrid Precoding for mmWave MU-MIMO Systems with Domestic Switch Network

**DOI:** 10.3390/s21093019

**Published:** 2021-04-25

**Authors:** Xiang Li, Yang Huang, Wei Heng, Jing Wu

**Affiliations:** National Mobile Communications Research Laboratory, Southeast University, Nanjing 210096, China; 230159390@seu.edu.cn (X.L.); 220190681@seu.edu.cn (Y.H.); 230169362@seu.edu.cn (J.W.)

**Keywords:** block diagonalization, cross-entropy, hybrid precoding, mmWave, MU-MIMO

## Abstract

Hybrid precoding is an attractive technique in MU-MIMO systems with significantly reduced hardware costs. However, it still requires a complex analog network to connect the RF chains and antennas. In this paper, we develop a novel hybrid precoding structure for the downlink transmission with a compact RF structure. Specifically, the proposed structure relies on domestic connections instead of global connections to link RF chains and antennas. Fixed-degree phase shifters provide candidate signals, and simple on-off switches are used to route the signal to antennas, thus RF adders are no longer required. Baseband zero forcing and block diagonalization are used to cancel interference for single-antenna and multiple-antenna users, respectively. We formulate how to design the RF precoder by optimizing the probability distribution through cross-entropy minimization which originated in machine learning. To optimize the energy efficiency, we use the fractional programming technique and exploit the Dinkelbach method-based framework to optimize the number of active antennas. Simulation results show that proposed algorithms can yield significant advantages under different configurations.

## 1. Introduction

Multiuser multiple-input multiple-output (MU-MIMO) is considered a key technology to meet the increasing traffic and critical energy efficiency demand in cellular networks [1,2]. The base station (BS) equipped with a large number of antennas can serve multiple users simultaneously. In a large-scale MIMO transmission scenario, the array gain is reaped to combat the severe path loss in millimeter wave (mmWave) channels by massive antennas at an order of a hundred or more [3]. Traditional fully-digital precoding structure requires a dedicated radio frequency (RF) chain consisting of a digital-to-analog converter (DAC), mixer, filter, power amplifier for each antenna [4]. This leads to unacceptable hardware costs as well as high energy consumption when plenty of transmit antennas are adopted in the massive MIMO scenario. Hybrid precoding involves a low-dimension digital precoder but preserves the high-dimension analog array at the same time [5,6,7]. It can be implemented to provide satisfactory rate performance and avoid a large number of RF chains than the fully-digital solutions. In the hybrid process, the analog beamformer is aimed at harvesting the array gain to improve the spectral efficiency when a digital baseband precoder is used to eliminate the inter-chain interference [8].

Hybrid precoding has been studied in much literature. In [9], the authors investigate the performance of two typical precoding architectures for hybrid precoding, namely fully-connected (FC) and sub-connected (SC). Numerical results show that the SC structure achieves comparable spectral efficiency performance with its counterpart but saves substantial hardware complexity and power consumption. Hybrid precoding based on orthogonal matching pursuit (OMP) is proposed in [4] by utilizing spatial sparsity. However, the predefined codebook limits the performance of the analog beamformer. By factorizing the equivalent channel matrix, near-optimal hybrid precoding algorithms for both FC and SC structure are proposed based on alternating minimization and then extended to the mmWave multi-carrier system in [10]. In [11], a deep neural network (DNN) is introduced as a framework to train the precoder and combiner, which achieves better performance compared with the conventional block diagonalization (BD). Hybrid precoding in terahertz communications is investigated in [12], where ultra-massive antennas are deployed to overcome the huge propagation loss. Then, a dynamic subarray hybrid precoding scheme is proposed to balance spectral efficiency and power consumption.

Matrix theory demonstrates that linear-digital precoding schemes, such as match filter (MF) [13], zero forcing (ZF) [14], and BD [15], can help promote the system throughput and simplify the precoder and combiner design for multiuser communication. ZF precoding, which cancels the inter-user interference through matrix inversion, is a practical scheme to be implemented because of the significantly simplified receiver design where mobile stations (MSs) no longer need a combining process [16]. However, hybrid ZF requires an equivalent number of RF chains as antennas at the MS. When MSs are equipped with extra antennas to promote their array gain, hybrid precoding and combining scheme should be designed to cope with cross-stream interference as well as inter-user interference. BD can be viewed as a generalization of the ZF to deal with this situation which uses singular value decomposition (SVD) as the matrix tool to eliminate the inter-chain interference and provides a closed-form solution to calculate the digital precoding and combining matrix [17]. The traditional BD achieves a sub-optimal capacity performance by selecting from the DFT set as the RF combiner and using equal gain transmission (EGT) as the RF precoder design criterion. The energy and spectral efficiency comparison of MU-MIMO systems with different linear processing schemes can be found in [18].

Energy efficiency is another issue when designing a hybrid process algorithm. The use of an SC structure can naturally reduce power consumption compared with the FC structure [19]. The power consumption can be reduced by implementing a low-resolution quantization for both the precoder and combiner [20]. Optimizing the number of RF chains also shows better energy efficiency than the conventional hybrid beamforming architecture with a 1-bit sampling resolution [21]. In most conventional hybrid processing structures, a large number of analog phase shifters (PSs) and RF adders are required to construct an RF precoder. To reduce the hardware complexity, especially the expensive high-resolution variable-degree PSs and multiple-input RF adders, a simplified hybrid precoding structure is proposed in [22] by introducing negative signals via RF inverters. Based on this switch-and-inverter (SI) based structure, an adaptive hybrid precoding algorithm is proposed by randomly generating near-optimal tests to improve spectral efficiency [23]. However, the major drawback of this structure is that it only provides two-phase candidates (0 and π) for each RF chain, and the binary cross-entropy (CE) minimization algorithm cannot be directly applied to multiphase optimization.

**Figure 1:**
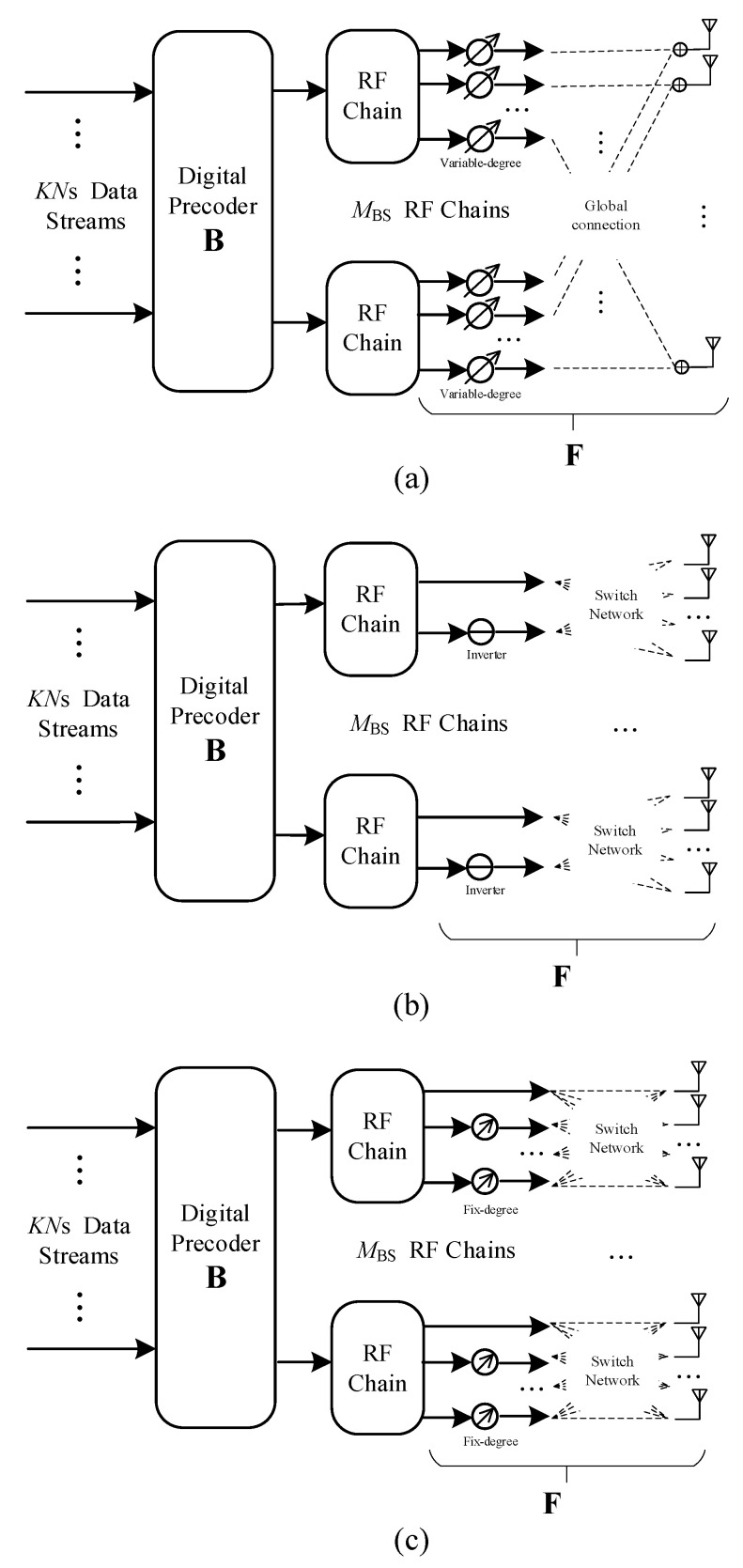
Hybrid precoder for MU-MIMO: (**a**) Fully-connected precoder. (**b**) Switch-inverter-based precoder. (**c**) Proposed hybrid precoder.

In this study, we consider the downlink transmission of the MU-MIMO system. As shown in Figure 1, a novel RF precoding structure is proposed by replacing the global connection with a domestic switch network (DSN). Compared with the SC structure, DSN uses fixed-degree PSs instead of variable-degree PSs to generate candidate signals, and the network is linked via simple on-off switches instead of RF adders. Therefore, our RF structure is more compact and consumes less energy than the SC hybrid precoder. CE is an iterative random sampling approach that is based on sampling and updating an underlying distribution function over the set of feasible solutions [24]. It has been widely used in ML systems to perform discrete optimization which is the core of our algorithm. The asymptotical convergence and convergence rate of the CE families have been discussed in several pieces of literature. In [25], the authors present theoretical convergence results for CE-based discrete optimization. Theoretical analysis and simulation results show that CE can find an optimal solution with a probability arbitrarily close to 1. In [26], the author proves that the optimal solution is reachable at some iteration. The idea and technique of this proof are inspired by the ant algorithm and simulated annealing, however, it fails to predict the convergence rate or residual error. In [27] authors give two conditions for a good convergence of CE in dealing with stochastic optimization. If relaxing any constraints, alternative improvements are proposed to guarantee good convergence. In [28] authors exploit the robust feature of CE by exploiting its similarity with the stochastic approximation procedure. It shows sure convergence in the Monte-Carlo simulation when a finite number of candidate solutions are generated at each iteration.

Inspired by CE, we propose a hybrid precoding algorithm in which candidate precoding matrices are generated according to a specified probability distribution, and the elite candidates are selected to update the distribution in each iteration. After several iterations, the distribution converges to a stable state, and the obtained precoder can be sufficiently close to the optimal. CE has shown potential sum-rate performance with its counterparts in recent studies [29], however, its searching space is significantly reduced than brute force. Two factors accelerate our algorithm to achieve a faster convergence rate than the classic CE. One is that we adopt a descending weight where samples with a large sum rate could have a higher contribution to the next state, while in classic CE all the elites have equal weights. Another is that classic CE implements a gradient descent algorithm with a constant smoothing parameter to update the sampling distribution, however, we derive the direct answer to find the optimal solution in our algorithm. Compared with the SI-based scheme, the proposed structure provides more phase candidates, thus a higher array gain can be achieved. The design of the analog precoder is formulated as a non-convex optimization problem. We adopt the CE with a weighted log-probability distribution as the metric to solve the problem, and a closed-form solution is derived to update the probability matrix in each iteration. The contributions of this study can be summarized as follows:

- A novel hybrid precoding structure based on DSN is proposed. We develop a CE-based framework to generate the hybrid precoding matrix in an iterative way.
- For single-antenna MSs, we extend the SI-based hybrid precoding algorithm to multiphase hybrid ZF. For multiple-antenna MSs, we formulate how to cancel inter-user and inter-stream interference via BD decomposition.
- We develop a Dinkelbach-method (DM) based algorithm to maximize the energy efficiency in which the number of active antennas is dynamically adapted.

The rest of this paper is organized as follows. Section 2 presents the system model of the downlink mmWave MU-MIMO system. In Section 3, a hybrid precoder with DSN is investigated for single-antenna and multi-antenna MSs. Energy efficiency optimization is studied in Section 4. Section 5 provides the simulation results and performance evaluation. Finally, the conclusion is drawn in Section 6.

Notation: Upper- and lower-case boldface denote matrices and vectors, respectively. E[·] represents the expectation and C represents the ensemble of complex numbers. **X**^H^, **X**^−1^, and ‖·‖_F_ denote the conjugate transpose, inversion and Frobenius norm of matrix **X**. Finally, **I***_N_* is the *N* × *N* identity matrix and **0** is the all-zero matrix.

## 2. System Model

### 2.1. System Model

We consider the downlink transmission of the MU-MIMO system. BS is equipped with $N_{\mathrm{BS}}$ antennas and *M*_BS_RF chains, and *K* fully-digital MSs are active simultaneously. Each MS has $N_{\mathrm{MS}}$ antennas and equivalent RF chains to support *N*s data streams, so there are totally *KN*s data streams to be scheduled at the BS. To guarantee the effectiveness of communications and maximize the system throughput with limited hardware resources, the number of RF chains should be constrained by *KN*s ≤ *M*_BS_ ≤ $N_{\mathrm{BS}}$ for the BS and *N*s ≤ *N*_MS_ for the MS.

At the BS, the transmitted symbol vector **s** is addressed by a fully-digital baseband precoder **B** of $M_{\mathrm{BS}}$× *KN*s and then an $N_{\mathrm{BS}}$× $M_{\mathrm{BS}}$ RF precoder **F**. Baseband precoder allows both amplitude and phase modification on the signal, while only phase shift can be realized by RF precoder via the analog circuit. We assume each entry of **F** has a unit amplitude and **B** is designed to meet the transmitted power limitation, i.e., ‖**FB**${\|}_{F}^{2}$ = *KN*s. We assume a non-frequency selective fading channel, the signal arrived at the *k*^th^ MS is

(1) $$y_{k}=H_{k}FBs+n_{k},$$

where **s** = [**s**_1_, **s**_2_, …, **s***_K_*]^T^ ∈ C*^KN^*^s*×*1^ is the vector that contains the signal of all the MSs. The signal vector satisfies E[**ss**^H^] = *P*/(*KN*s)**I***_KN_*_s_, where *P* is the average transmit power. $H_{k}$ ∈ C*^N^*^${}_{\mathrm{MS}}{\times N}_{\mathrm{BS}}$^ is the channel matrix of the *k*^th^ MS, and $n_{k}$ is the $N_{\mathrm{MS}}$ × 1 i.i.d additive noise vector obeying CN(0, σ^2^).

The *k*^th^ MS processes the received signal by digital combining as

(2) $${\hat{x}}_{k}=M_{k}^{H}y_{k}=M_{k}^{H}H_{k}FBs+M_{k}^{H}n_{k},$$

where **M***_k_* is the *N*_MS_ × *N*s baseband combiner for the *k*^th^ MS. To simplify the analysis, the equivalent baseband channel of *k*^th^ MS is defined as

(3) $${\tilde{H}}_{k}=H_{k}F.$$

Then the signal processing at the *k*^th^ MS can be rewritten as

(4) $${\hat{x}}_{k}=M_{k}^{H}{\tilde{H}}_{k}B_{k}s_{k}+\underset{\mathrm{interference}}{\underbrace{\sum_{i=1,i\neq k}^{K}M_{k}^{H}{\tilde{H}}_{k}B_{i}s_{i}}}+\underset{\mathrm{noise}}{\underbrace{\overset{}{\underset{{}_{}}{\;M_{k}^{H}n_{k}\;}}}}$$

in which **B***_k_* is the baseband precoding matrix corresponding to the *k*^th^ MS, i.e., the ((*k* − 1)*N*s + 1)^th^~(*kN*s)^th^ columns of **B**. Assuming the modulated symbols are Gaussian, the sum spectral efficiency can be expressed as

(5) $$R=\sum_{k=1}^{K}\log_{2}\left(\left|I_{Ns}+SINR_{k}\right|\right),$$

where **SINR***_k_* is the overall signal-to-interference-plus-noise ratio of the *k*^th^ MS.

### 2.2. Channel Model

MmWave channel has different propagation characteristics when compared with the low-frequency channels. It no longer obeys the conventional Rayleigh fading [30] and clustered mmWave channel model can be used to characterize the limited scattering feature of the mmWave channel. The normalized mmWave downlink channel of the *k*th MS is modeled as the sum of all the propagation paths which is scattered in *N*_C_ clusters and each cluster involves total *N*_P_ paths. It can be expressed as

(6) $$H_{k}=\sqrt{\frac{1}{N_{C}N_{P}}}\sum_{c=1}^{N_{C}}\sum_{p=1}^{N_{P}}g_{c,p}^{k}a_{\mathrm{MS}}\left(\theta_{c,p}^{k}\right)a_{\mathrm{BS}}{\left(\varphi_{c,p}^{k}\right)}^{H}$$

in which $g_{c,p}^{k}$ corresponds to the complex channel gain of the *p*^th^ path of the *c*^th^ cluster. $\theta_{c,p}^{k}$ and $\varphi_{c,p}^{k}$ are the azimuth angle of the arrival and departure (AoA and AoD), respectively. **a**_MS_($\theta_{c,p}^{k}$) and **a**_BS_($\varphi_{c,p}^{k}$) are the corresponding receive and transmit array response vectors while the elevation dimension is ignored. In each cluster, we assume $\theta_{c,p}^{k}$ and $\varphi_{c,p}^{k}$ are distributed within a specified range which is generated by truncated Laplacian distribution.

Without loss of generality, we chose the uniform linear array (ULA) to model the BS and MSs array in this study. For an *N* antenna array, the response vector is given as

(7) $$a\left(\theta \right)=\frac{1}{\sqrt{N}}{\left[1,\;e^{j2\pi d\sin(\theta )/\lambda },\;\ldots ,\;e^{j2\pi (N-1)d\sin(\theta )/\lambda }\right]}^{T},$$

where *λ* is the wavelength of the carrier frequency and *d* is the distance between the adjacent antennas. Assuming a half-wavelength antenna spacing in this study, the array response of BS or MS can be calculated from Equation (7). Furthermore, other antenna array patterns can also be adopted and the proposed scheme can be directly applied to arbitrary antenna arrays. Due to the sparse nature of the mmWave, the channel coefficients can be effectively estimated by compressing sensing algorithms [31,32]. We assume **H***_k_* is known to the BS, and all the MSs have the full information of their combining matrix.

### 2.3. Hybrid Precoder

As shown in Figure 1a, in the broadly discussed FC hybrid precoder, each RF chain is connected to all antennas through variable-degree PSs and RF adders. It can approach the performance of the unconstrained maximization [33]. In the FC structure, the RF precoding matrix should have a format as

(8) $$F=\left[\begin{array}{ccc} f_{1,1} & \cdots & f_{1,M_{\mathrm{BS}}}\\ \vdots & \ddots & \vdots \\ f_{N_{\mathrm{BS}},1} & \cdots & f_{N_{\mathrm{BS}},M_{\mathrm{BS}}} \end{array}\right].$$

Hybrid precoding also adds constraints to the RF precoder where only *B*-bit quantized shift can be applied to each element, i.e.,

(9) $$f^{\left(p,q\right)}\in \left\{1,\;e^{j\frac{2\pi }{2^{B}}},\;\ldots \;,\;e^{j\frac{2\pi \times (2^{B}-1)}{2^{B}}}\right\},$$

where *f*^(*p*,*q*)^ is the (*p*, *q*)^th^ element of **F**. It has been considered in many studies to achieve full precoding gain [9,10,11,12,13,14,15,16,17,18,19,20,21,22,23,24,25,26,27,28,29,30,31,32,33,34]. Most of these algorithms decompose the problem into analog precoder design and digital precoder design separately. Specifically, the analog precoder **F** is first generated to maximize the sum-rate according to the channel matrix **H***_k_* or selected from a predefined codebook. After that, $H_{k}$**F** can be viewed as the equivalent channel coefficient, and the digital precoder is designed to eliminate inter-chain interference.

To cut down on the hardware complexity, one feasible solution is to replace the FC RF network with a switch-based SC structure [34] in which each RF chain is only connected to *M* antennas rather than all antennas via switches where *M* = *N*_BS_/$M_{\mathrm{BS}}$. However, due to the elementary on-off connection, this structure fails to realize the full array gain. As shown in Figure 1b, an RF inverter is introduced after each RF chain which provides a negative signal [22], so a higher achievable sum rate can be achieved. The format of this RF precoding matrix can be written as

(10) $$F=\left[\begin{array}{cccc} f_{1} & 0 & \cdots & 0\\ 0 & f_{2} & \cdots & 0\\ \vdots & \vdots & \ddots & \vdots \\ 0 & 0 & \cdots & f_{M_{\mathrm{BS}}} \end{array}\right],$$

where **f***_m_* denotes the analog precoding vector of the *m*^th^ RF chain with size *M* × 1 and each element should be in the set {±1}. Equation (10) is a block diagonal matrix and the off-diagonal elements are zero because there is no cross-chain connection in our structure. The effectiveness of this structure has been proven in theory and practice.

As shown in Figure 1c, the proposed precoder evolves the SI-based by introducing an additional phase-shifted signal. The constrain on the entries of **F** can be rewritten as

(11) $$f^{\left(p,q\right)}\in \left\{1,\;e^{j\frac{2\pi }{L}},\;\ldots \;,\;e^{j\frac{2\pi \times (L-1)}{L}}\right\},$$

where the phase-shifted signal can be pregenerated by *L*−1 fixed-degree PSs, and the switch network routes the signal to each antenna.

In a typical design procedure, the optimization of the precoder should be an essential method to achieve a maximal sum rate or minimal distance to the optimal precoder. Finding the spectral efficiency precoding matrix are non-coherent combining optimization problems [35], and the large-scale precoding matrix makes it almost intractable to find the global optimum through exhaust search while maintaining the constraints imposed on the precoder [4]. Even in the fully-digital MU-MIMO systems, it still requires enormous efforts to find a local optimum of sum rate [36].

## 3. Hybrid Precoding

With a large number of antennas deployed in the considered scenario, array gain should be cultivated through the appropriate designed switch-only network. Two conditions should be met at the same time: one is that the equivalent channel should be well-conditioned enough to support reliable transmission of up to *KN*s streams; another is that after decomposing the equivalent channel, spectral efficiency should be as larger as possible. We formulate how to design the precoder for hybrid-ZF and hybrid-BD precoding in this section.

### 3.1. Hybrid-ZF and Array Gain Harvesting

Based on the structure proposed in the previous section, the purpose of the proposed hybrid-ZF precoder is to maximize *R* with respect to **F** and **B**. It can be described as

(12) $$\begin{array}{c} \left\{F,B\right\}=\underset{F,B}{\arg\max}\;R=\underset{F,B}{\arg\max}\sum_{k=1}^{K}\log_{2}\left(1+\gamma_{k}\right)\;\\ \begin{array}{l} \mathrm{subject}\;\mathrm{to}\;F\in F,\\ \;\;\;\;\;\;\;\;\;\;\;\;\;\;\;\;{\left\|FB\right\|}_{F}^{2}=KNs, \end{array} \end{array}$$

in which F is the set of all the possible analog precoding matrices satisfying Equations (10) and (11). The equivalent channel is

(13) $$H_{\mathrm{eq}}={\left[H_{1}^{T},\;\ldots ,H_{K}^{T}\right]}^{T}F,$$

and for ZF digital precoder, **B** can be calculated by

(14) $$B=\frac{\sqrt{KNs}{\left(H_{\mathrm{eq}}\right)}^{H}{\left(H_{\mathrm{eq}}{\left(H_{\mathrm{eq}}\right)}^{H}\right)}^{-1}}{{\left\|F{\left(H_{\mathrm{eq}}\right)}^{H}{\left(H_{\mathrm{eq}}{\left(H_{\mathrm{eq}}\right)}^{H}\right)}^{-1}\right\|}_{F}^{2}}.$$

Then the SINR of the *k*^th^ user *γ_k_* can be calculated as

(15) $$\gamma_{k}=\frac{{\left\|H_{k}Fb_{k}\right\|}_{F}^{2}}{\sigma_{k}^{2}+\sum_{i\neq k}{\left\|H_{k}Fb_{i}\right\|}_{F}^{2}},$$

where **b***_k_* is the *k*^th^ column of the digital baseband precoder **B**. 

The core of hybrid-ZF precoding is to design **F** under given constraints. One solution is the exhaustion search: **F** has *N*_BS_ non-zero elements and each element have *L* potential values, so there are $L^{N_{\mathrm{BS}}}$ possibilities. However, in the massive MIMO configuration, *N*_BS_ is usually very large, so finding the global optimum while maintaining the constraints imposed on the RF precoder is often computationally intractable. In the proposed algorithm, we resort to iteratively generating near-optimal tests and optimizing the distribution by minimizing the cost function in a closed-form. Specific steps are detailed as follows:

**Step I**, initializing the equal-probable matrix **P**^1^ whose element $p_{l,n}^{1}$ indicates the possibility of the *l*^th^ phase of the *n*^th^ non-zero element in **F** where 1 ≤ *l* ≤ *L* and 1 ≤ *n* ≤ *N*_BS_.

**Step II**, according to **P***^m^*, where *m* represents the iteration index, randomly generating *S* individuals **F***^s^*, where 1 ≤ *s* ≤ *S*, and computing the corresponding **B***^s^* by Equations (13) and (14).

**Step III**, calculating the sum rate *R^s^* by Equations (12) and (15) before sorting {*R^s^*} in a decent order as {*R*_1_, *R*_2_, …, *R_S_*}. Then we pick the *E* largest as the elite-batch.

**Step IV**, computing the CE using the weighted log-probability distribution as

(16) $$\varepsilon \left(P^{m}\right)=\sum_{e=1}^{E}w_{e}\sum_{n=1}^{N}\sum_{l=1}^{L}\delta_{e,n,l}^{m}\ln p_{n,l}^{m},$$

where *δ_e,n,l_* is the binary activity indicator. Only when the *l*^th^ phase candidate of the *n*^th^ antenna is selected at *e*^th^ test, *δ_e,n,l_* is active. *w_e_* is the weight of the elite-batch defined by

(17) $$w_{e}=\frac{\left|R_{e}-R_{E}\right|}{\sum_{e=1}^{E}\left|R_{e}-R_{E}\right|}\;\;\;\left(1\leq e\leq E\right).$$

**Step V**, updating the probability matrix following

(18) $$P^{m+1}=\underset{P^{m}}{\arg\min}\varepsilon \left(P^{m}\right).$$

The problem can be formulated as

(19) $$\begin{array}{c} \mathrm{minimize}\;\;\varepsilon \left(P^{m}\right)\\ \mathrm{subject}\;\mathrm{to}\;\sum_{l=1}^{L}p_{n,l}^{m}=1,\;\mathrm{for}\;n=1,\ldots ,N_{\mathrm{BS}}. \end{array}$$

We introduce multipliers *ε_n_* where *n* = 1, …, *N*_BS_, the Lagrange function can be constructed as

(20) $$ℒ\left(P^{m},\varepsilon_{1},\ldots ,\varepsilon_{N}\right)=\sum_{e=1}^{E}w_{e}\sum_{n=1}^{N_{\mathrm{BS}}}\sum_{l=1}^{L}\delta_{e,n,l}^{m}\ln p_{n,l}^{m}-\;\sum_{r=1}^{N_{\mathrm{BS}}}\varepsilon_{r}\left(\sum_{l=1}^{L}p_{l,n}-1\right),$$

and solving totally (*N*_BS_*L* + *N*_BS_) equations

(21) $$\begin{array}{l} \;\;\;\;\;\nabla_{p_{n,l}^{m},\;\varepsilon_{r}}ℒ\left(P^{m},\varepsilon_{1},\ldots ,\varepsilon_{N}\right)=0,\;\\ \mathrm{where}\;1\leq l\leq L,\;1\leq n\leq N_{\mathrm{BS}},\;1\leq r\leq N_{\mathrm{BS}}. \end{array}$$

Finally, we have

(22) $$p_{n,l}^{m+1}=\frac{\sum_{e=1}^{E}w_{e}\delta_{e,n,l}^{m}}{\sum_{e=1}^{E}\sum_{l=1}^{L}w_{e}\delta_{e,n,l}^{m}},\;\mathrm{for}\;1\leq l\leq L,\;1\leq n\leq N_{\mathrm{BS}}.$$

**Step VI**, let *m*←*m* + 1 and restart the loop from step II until *w_e_* = 0 for all *e* or reaches the maximum number of iterations. Finally, we output **F***^opt^* and **B***^opt^* which have the largest sum rate *R^opt^*.

The complete process of the proposed algorithm is listed in Algorithm 1. In the proposed algorithm, *w_e_* in Equation (17) create the unfairness for each elite to accelerate the convergency and provides a simple ending criterion in Step VI.
**Algorithm 1**: Proposed hybrid-ZF algorithm**Input: H**, σ^2^1:  **Loop**:2:   **for**
*s*
**in** 1…*S*
**do**
3:    Generate **F**^s^ according to **P***^m^*;4:    Calculate **B**^s^ and *R*^s^ by Equations (12)–(15);5:   **for**
*e*
**in** 1…*E*
**do**
6:    Calculate *w_e_* by Equation (17);7:   **end for**8:   Check convergency; 9:   **for**
*n*
**in** 1…*N*_BS_
**do**
10:    **for**
*l*
**in** 1…*L*
**do**
11:     Update $p_{l,n}^{m+1}$ by Equation (22); 12:   **end for**13:   **end for**14:   *m*←*m* + 1;15:  **end loop**16: **return F**^opt^, **B**^opt^ and *R^opt^*.

### 3.2. Hybrid Block Diagonalization

For multiple-antenna MSs, the goal of hybrid-BD is to maximize *R* with respect to **F**, **B**, and $M_{k}$, where 1 ≤ *k* ≤ *K*. It can be described as

(23) $$\begin{array}{c} \left\{F,B,M_{k}\right\}=\underset{F,B,\;M_{k}}{\arg\max}\;\sum_{k=1}^{K}\log_{2}\left(\left|I_{Ns}+{\mathrm{SIN}R}_{k}\right|\right)\;\\ \begin{array}{l} \mathrm{subject}\;\mathrm{to}\;F\in F,\\ \;\;\;\;\;\;\;\;\;\;\;\;\;\;\;\;{\left\|FB\right\|}_{F}^{2}=KNs. \end{array} \end{array}$$

and the **SINR***_k_* is defined as

(24) $$SINR_{k}={\left(\sum_{i=1,i\neq k}^{K}M_{k}^{H}{\tilde{H}}_{k}B_{i}B_{i}{\tilde{H}}_{k}^{H}M_{k}+\sigma^{2}KNs/P\times M_{k}^{H}M_{k}\right)}^{-1}M_{k}^{H}{\tilde{H}}_{k}B_{k}B_{k}{\tilde{H}}_{k}^{H}M_{k}.$$

After generating the analog precoding matrix, we design the baseband BD precoder and combiner with available channel information. The baseband BD is designed to eliminate the inter-stream interference by forcing cross-stream terms zero. The entire equivalent baseband channel is denoted as

(25) $$H_{\mathrm{eq}}={\left[{\tilde{H}}_{1},\;{\tilde{H}}_{2},\ldots ,\;{\tilde{H}}_{K}\right]}^{T}={\left[H_{1}^{T},\;H_{2}^{T},\ldots ,\;H_{K}^{T}\right]}^{T}F.$$

The first step of baseband BD is to cancel the inter-user interference, i.e., ${\bar{H}}_{k}B_{i}=0$, for *k* ≠ *i*. Then the SINR of the *k*^th^ user can be written as

(26) $$SINR_{k}=P/\sigma^{2}KNs\times {\left(M_{k}^{H}M_{k}\right)}^{-1}M_{k}^{H}{\tilde{H}}_{k}B_{k}B_{k}{\tilde{H}}_{k}^{H}M_{k}.$$

To obtain **B**, we first define ${\bar{H}}_{k}$ as

(27) $${\bar{H}}_{k}={\left[{\tilde{H}}_{1}^{T},\ldots ,{\tilde{H}}_{k-1}^{T},{\tilde{H}}_{k+1}^{T},\ldots ,{\tilde{H}}_{K}^{T}\right]}^{T}.$$

Then **B***_k_* should lie in the null space of ${\bar{H}}_{k}$. The SVD of ${\bar{H}}_{k}$ is given by

(28) $${\bar{H}}_{k}={\bar{U}}_{k}{\bar{\Sigma }}_{k}{\left[{\bar{V}}_{k}^{\left(\left(K-1\right)\times N_{\mathrm{MS}}\right)},{\bar{V}}_{k}^{\left(N_{\mathrm{MS}}\right)}\right]}^{H}.$$

The rank of ${\bar{H}}_{k}$ is no larger than (*K* − 1) × $N_{\mathrm{MS}}$, so ${\bar{V}}_{k}^{\left(K-1\right)\times N_{\mathrm{MS}}}$ contains the largest (*K* − 1) × *N*_MS_ right singular vector and ${\bar{V}}_{k}^{\left(N_{\mathrm{MS}}\right)}$ has the rest singulars which is the orthogonal bases of the nulling space naturally. Therefore, we have

(29) $${\tilde{H}}_{i}^{T}{\bar{V}}_{k}^{\left(N_{\mathrm{MS}}\right)}=\{\begin{array}{cc} 0 & i\neq k\\ {\tilde{H}}_{k}^{T}{\bar{V}}_{k}^{\left(M_{\mathrm{MS}}\right)} & i=k \end{array}.$$

The block diagonalization at the precoder should be

(30) $$\begin{array}{l} H_{\mathrm{BD}}=H_{\mathrm{eq}}\left[{\bar{V}}_{1}^{\left(N_{\mathrm{MS}}\right)},\ldots ,{\bar{V}}_{K}^{\left(N_{\mathrm{MS}}\right)}\right]\\ \;\;\;\;\;\;=\left[\begin{array}{cccc} {\tilde{H}}_{1}{\bar{V}}_{1}^{\left(N_{\mathrm{MS}}\right)} & 0 & \ldots & 0\\ 0 & {\tilde{H}}_{2}{\bar{V}}_{2}^{\left(N_{\mathrm{MS}}\right)} & \cdots & 0\\ \vdots & \vdots & \ddots & \vdots \\ 0 & 0 & \cdots & {\tilde{H}}_{K}{\bar{V}}_{K}^{\left(N_{\mathrm{MS}}\right)} \end{array}\right], \end{array}$$

which creates the inter-user interference-free subchannels for each MS. Given the above results, all the MS could enjoy their independent transmission.

To maximize the spectral efficiency of the whole system, further decomposition should be performed to cancel inter-stream interference at the *k*^th^ user by

(31) $${\tilde{H}}_{k}{\bar{V}}_{k}^{\left(N_{\mathrm{MS}}\right)}=U_{k}\Sigma_{k}V_{k}^{H}.$$

${\tilde{H}}_{k}{\bar{V}}_{k}^{\left(N_{\mathrm{MS}}\right)}$ is a $N_{\mathrm{MS}}$ × $N_{\mathrm{MS}}$ square matrix, so totally $N_{\mathrm{MS}}$ subchannel allows *N*s data streams for each user as *N*s ≤ *N*_MS_. Therefore, the optimal precoder and combiner for the *k*^th^ user are $V_{k}^{\left(Ns\right)}$ and $U_{k}^{\left(Ns\right)}$, respectively.

Overall, the baseband precoder is given by combining the matrix as

(32) $$\begin{array}{l} B=\left[{\bar{V}}_{1}^{\left(N_{\mathrm{MS}}\right)},\ldots ,{\bar{V}}_{K}^{\left(N_{\mathrm{MS}}\right)}\right]\left[\begin{array}{ccc} V_{1}^{\left(Ns\right)} & \cdots & 0\\ \vdots & \ddots & \vdots \\ 0 & \cdots & V_{K}^{\left(Ns\right)} \end{array}\right]\\ \;\;\;=\left[{\bar{V}}_{1}^{\left(N_{\mathrm{MS}}\right)}V_{1}^{\left(Ns\right)},\ldots ,{\bar{V}}_{K}^{\left(N_{\mathrm{MS}}\right)}V_{K}^{\left(Ns\right)}\right], \end{array}$$

and the baseband combiner of the *k*^th^ user is given by

(33) $$M_{k}=U_{k}^{\left(Ns\right)}.$$

The sum-rate after hybrid BD can be expressed as

(34) $$R=\sum_{k=1}^{K}\log_{2}\left(\left|I_{Ns}+\frac{P{\left(\Sigma_{k}^{\left(Ns\right)}\right)}^{2}{\left(M_{k}^{H}M_{k}\right)}^{-1}}{\sigma^{2}KNs}\right|\right),$$

where $\Sigma_{k}^{\left(Ns\right)}$ is the left-top *N*s × *N*s submatrix of $\Sigma_{k}$. Since the combining matrix satisfies $M_{k}^{H}$ $M_{k}$= **I***_N_*_s_, Equation (34) can be simplified as

(35) $$R=\sum_{k=1}^{K}\log_{2}\left(\left|I_{Ns}+\frac{P{\left(\Sigma_{k}^{\left(Ns\right)}\right)}^{2}}{\sigma^{2}KNs}\right|\right).$$

After the above hybrid-BD process, interference-free transmission between each stream is achieved. Then the CE can be computed using the weighted log-probability distribution and the probability matrix can be updated through CE minimization.

However, equal power allocation is not enough for optimizing the sum rate. In the next subsection, a water-filling algorithm is adopted to maximize the spectral efficiency by matching the channel gain.

### 3.3. Water-Filling Power Allocation

In this section, we formulate the optimal power allocation for each stream to maximize the sum-rate by the water-filling algorithm. Our purpose is to find **Λ** such that

(36) $$\begin{array}{l} \Lambda \;=\;\underset{\Lambda }{\mathrm{argmax}}R\\ \;\;\;=\underset{\Lambda }{\mathrm{argmax}}\sum_{k=1}^{K}\log_{2}\left(\left|I_{Ns}+\frac{P\Lambda_{k}\eta_{k}^{\left(Ns\right)}{\left(\Sigma_{k}^{\left(Ns\right)}\right)}^{2}}{\sigma^{2}KNs}\right|\right)\\ \;\mathrm{subject}\;\mathrm{to}:\;\mathrm{trace}\left(\Lambda \right)=KNs,\\ \;\;\;\;\;\;\;\;\;\;\;\;\;\;\;\;\;\;\;\;\;\;\;\;\mathrm{diag}\left(\Lambda \right)\geq 0. \end{array}$$

where $\eta_{k}^{\left(Ns\right)}$ is the *N*s × *N*s diagonal matrix for the *k*^th^ MS which contains the positive weight for each stream. **Λ** is defined by

(37) $$\Lambda =\left[\begin{array}{ccc} \Lambda_{1} & \cdots & 0\\ \vdots & \ddots & \vdots \\ 0 & \cdots & \Lambda_{K} \end{array}\right]=\left[\begin{array}{ccc} \Delta_{1} & \cdots & 0\\ \vdots & \ddots & \vdots \\ 0 & \cdots & \Delta_{KNs} \end{array}\right]$$

as the *KN*s × *KN*s power allocation matrix, in which Δ*_k_* is its diagonal elements. Denoting *γ_n_* as the *n*^th^ diagonal element of *P*/(σ^2^*KN*s) × diag{$\Sigma_{1}^{\left(Ns\right)}$, $\Sigma_{2}^{\left(Ns\right)}$, …, $\Sigma_{K}^{\left(Ns\right)}$}, i.e., the channel gain of the *n*^th^ stream, the problem can be rewritten in a classical convex optimization problem as

(38) $$\begin{array}{l} \underset{\left\{\Delta_{n}\right\}}{\arg\min}\;-\sum_{n=1}^{KNs}\eta_{n}\ln\left(1+\gamma_{n}\Delta_{n}\right)\\ \mathrm{subject}\;\mathrm{to}\;\sum_{n=1}^{KNs}\Delta_{n}=KNs,\\ \;\;\;\;\;\;\;\;\;\;\;\;\;\;\;\;\Delta_{n}\geq 0,\;\mathrm{for}\;n\;\mathrm{in}\;1,\ldots ,KNs. \end{array}$$

As indicated in [37], real Lagrange multipliers {$m_{1}$, …, $m_{KNs}$} are introduced for the inequality constraints Δ*_n_* and a real multiplier *v* for the equality constraints, the KKT conditions are

(39) $$\begin{array}{l} \sum_{n=1}^{KNs}\Delta_{n}=KNs,\;\Delta_{n}\geq 0,m_{n}\geq 0,\Delta_{n}m_{n}=0,\\ -\frac{\eta_{n}\gamma_{n}}{1+\gamma_{n}\Delta_{n}}-m_{n}+v=0,\;\mathrm{for}\;n=1,\ldots ,KNs. \end{array}$$

We have the intermediate results that

(40) $$\Delta_{n}m_{n}=\Delta_{n}\left(v-\frac{\eta_{n}\gamma_{n}}{1+\gamma_{n}\Delta_{n}}\right)=0,\;$$

and finally, we have

(41) $$\Delta_{n}=\max\left\{\frac{\eta_{n}}{v}-\frac{1}{\gamma_{n}},0\right\}.\;$$

The solution revises the classical water-filling power allocation algorithm where the adjustable stream priority *η_n_* is taken into consideration to guarantee a reliable transmission of up to *KN*s streams.

The complete process of the proposed hybrid-BD algorithm is listed in Algorithm 2. Note that proposed algorithms do not rely on any precondition of the channel matrix. It can be adopted in the mmWave sparse scattering channel as well as the typical i.i.d Rayleigh channel as long as the channel matrix is available.
**Algorithm 2**: Proposed hybrid-BD algorithm **Input**: **H***_k_*, σ^2^1:  **Loop**:2:  **for**
*s*
**in** 1,…,*S*
**do**
3:   Generate **F***^s^* according to **P***^m^*;4:   Calculate **B***^s^* and $M_{k}^{s}$ by Equations (32) and (33);5:   Calculate Δ*_n_* by Equation (41) and the corresponding spectral efficiency *R^s^*;6:  **end for**7:  **for**
*e*
**in** 1,…,*E*
**do**8:   Calculate the weight *w_e_*;9:  **end for**10:  Check convergency;11:  **for**
*n*
**in** 1,…,*N*_BS_
**do**
12:    **for**
*l*
**in** 1,…,*L*
**do**
13:     Update
$p_{l,n}^{m+1}$14:   **end for**15:  **end for**16:  *m*←*m* + 1;17:  **end loop**18:   **return**
**F***^opt^*, **B***^opt^*, and $M_{k}^{opt}$

## 4. Energy Efficiency Maximization

In this section, we derive the approach that aims at maximizing energy efficiency by dynamic antenna selection. In terms of achievable sum-rate *R* and consumed power *P*, the energy-efficient ratio for the hybrid precoding is defined as

(42) $$\mathrm{EE}\left(F\right)≜\frac{R\left(F\right)}{P\left(F\right)}\;\left(\mathrm{bits}/\mathrm{Hz}/J\right),$$

where *R*(·) represents the information rate in bits/Hz/J and *P*(·) is the consumed power in watt. We adopt the power consumption model proposed in [34], where the power consumption of the DSN can be expressed as

(43) $$P=N_{\mathrm{BS}}\times P_{\mathrm{PA}}+\left(L-1\right)M_{\mathrm{BS}}\times P_{\mathrm{PS}}+KNs\times \left(P_{\mathrm{RFC}}+P_{\mathrm{DAC}}\right)+P_{\mathrm{BB}}.$$

*P*_PA_, *P*_PS_, *P*_RFC_, and *P*_DAC_ are the power of the power amplifier, phase shffter, RF chain, and DAC respectively. *P*_BB_ denotes the power of digital signal processing. It can be observed from Equation (43) that DSN requires less power on *P*_PS_ than the FC structure which contains *N*_BS_ × *M*_BS_ PSs and the power of RF adders is omitted. The values of each parameter employed in this work are set as follows: *P*_PA_ = 20 mW, *P*_PS_ = 30 mW, *P*_RFC_ = 30 mW, *P*_DAC_ = 200 mW, and *P*_BB_ = 5 mW [34]. Note that if the fixed-degree phase shift is implemented by a passive component, e.g., delay line [38], its power consumption can be ignored.

To explain the antenna selection mechanism, we add an extra candidate to the possible phases, e.g., 0, which represents an open switch of the antenna, while the non-zero values still determine the phase of the corresponding active antenna. Increasing the number of active antennas, we might have a higher information rate but there is also higher power consumption. The problem of maximizing Equation (42) can be solved by fractional programming theory. Among the possible options, DM is a computationally efficient solution [39]. DM replaces the fractional cost function of Equation (42) into iteratively solving the difference-based problems. The cost function can be rewritten by

(44) $$\Phi \left(F^{\left(m\right)},\;\psi^{\left(m\right)}\right)=R\left(F^{\left(m\right)}\right)-\psi^{\left(m\right)}P\left(F^{\left(m\right)}\right),$$

where *ψ*^(*m*)^ = *R*(**F**^(*m*-1)^)/*P*(**F**^(*m*-1)^) ∈ $R^{+}$, and *m* denotes the iteration index. In each loop, the ratio *ψ*^(*m*)^ is updated based on the previously achieved rate and power consumption. It requires re-running Algorithm 1 or 2 as the inner loop to approximate the optimal power scheme whose detail procedure is driven by the CE minimization. The pseudo-code of the DM-based energy efficiency optimization algorithm is provided in Algorithm 3.
**Algorithm 3**: DM-based energy efficiency optimization1:  Calculate *R*(**F**^(0)^) via Algorithm I or II and corresponding *P*(**F**^(0)^). 2:  Initialize *ψ*^(0)^ = *R*(**F**^(0)^) / *P*(**F**^(0)^), m = 0.3:  **while** Φ(**F**^(*m*)^, *ψ*^(*m*)^) ≥ 0 **do**4:  *m* = *m* + 1.5:  Maximize Φ(**F**^(*m*)^, *ψ*^(*m*)^) via CE minimization.6:  Update *ψ*^(*m*)^ and Φ(**F**^(*m*)^, *ψ*^(*m*)^).7:  **if** Φ(**F**^(*m*)^, *ψ*^(*m*)^) < 08:   break;9:  **end if**10:  **end while**
11:  **return F**^(*m*)^ and *ψ*^(*m*)^.

## 5. Simulation Results

In this section, we evaluate the achievable spectral and energy efficiency of the proposed algorithms with DSN by comparing it with the different hybrid-precoding schemes in typical MU-MIMO configuration. We model the mmWave propagation channel with *N*_C_ = 8 clusters and each cluster involves *N*_P_ = 10 paths. The angle spread of *θ^k^* and *φ^k^* are both equal to 7.5° and each path factor follows a complex Gaussian distribution with zero mean and unit variance. For all the scenarios, we have *M*_BS_= *KN*s. We set all the streams to have unit importance in the water-filling procedure and the SNR is defined as *P*/σ^2^.

### 5.1. Performance of Proposed Hybrid-ZF

In this subsection, we show the comparison of the achievable sum-rate against SNR of proposed hybrid-ZF, fully-digital ZF, quantized hybrid precoding [40], SI-based algorithm [23], and antenna selection (AS) [34]. Fully-digital ZF, quantized hybrid precoding, and AS are non-iterative algorithms. The parameters of the SI-based and proposed algorithm are set as follows: *S* = 500, *E* = 0.15*S*, and the maximum number of iterations are 20. The number of antennas *N*_BS_ is set to 64, and a total of 4 users are supported simultaneously.

**Figure 2:**
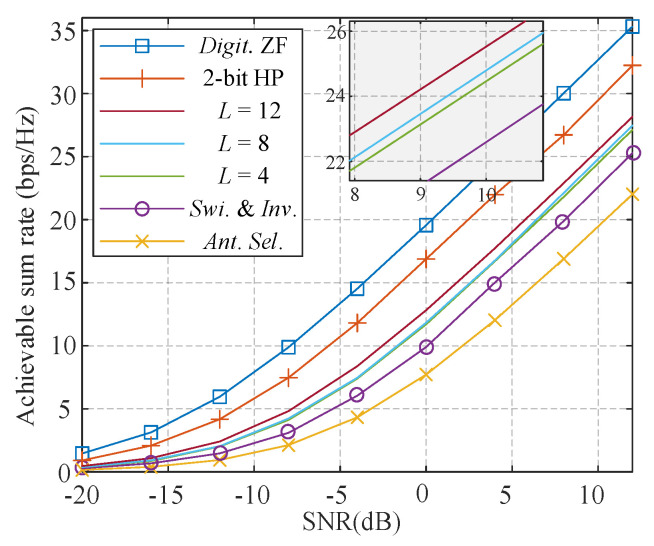
Achievable sum rate of different schemes.

As observed from Figure 2, Digital ZF provides the upper bound for all algorithms, and a 2-bit quantized hybrid precoder is also presented as a reference. Quantized hybrid precoding introduces 2 dB loss when compared with the digital ZF. AS has the poorest performance among all the schemes because it has the lowest spatial utilization and SI-based algorithm improves 3 dB compared with AS. Through the introduction of 3 fixed-degree PSs, the proposed algorithm with *L* = 4 is about 2 dB superior to the SI-based scheme and 11 PSs could further improve by 1 dB over a wide range of SNR.

**Figure 3:**
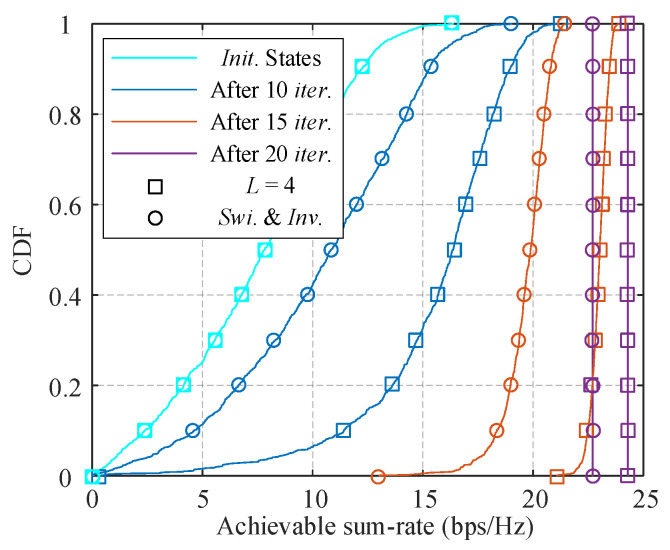
CDF of the achievable sum rate.

Figure 3 shows the achievable sum-rate cumulative distribution function (CDF) curves of proposed algorithm with *L* = 4 and SI-based algorithm when *N*_BS_ = 64, *K* = *M*_BS_ = 4, and SNR equals 10 dB. The simulations start from the same initial population and the multiphase superiority helps the proposed algorithm promote the performance much faster than that of the SI-based algorithm after the first 10 loops. As the generation evolves, the range of achievable sum-rate continues narrowing and the curve becomes steeper. After 20 loops, both the algorithms achieve their convergency as the weight decreases to zero, i.e., *R_e_* = *R_E_* for 1 ≤ *e* ≤ *E*, and the probability evolution terminated. Finally, the proposed algorithm advances the SI-based algorithm about 2 bps/Hz.

**Figure 4:**
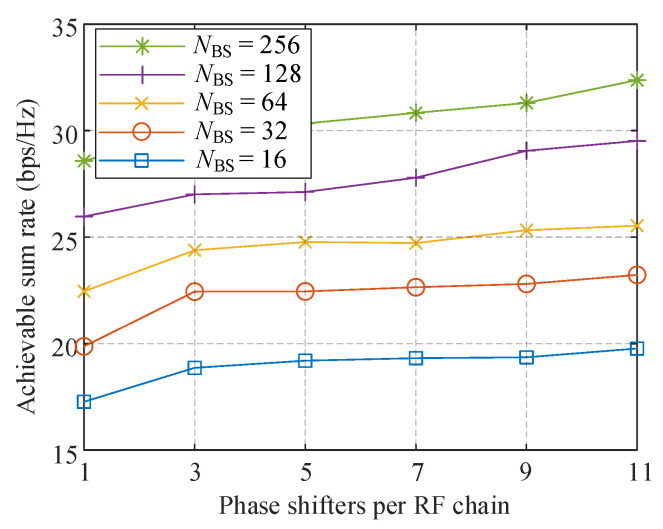
Comparison of achievable sum-rate with different *N*_BS_ against the number of PSs per RF chain.

In Figure 4, we show the performance comparison of the proposed hybrid-ZF with different RF antennas as a function of the number of fixed-degree PSs per RF chain. The parameters are set as follows: *K* = 4, *M*_BS_ = 4, and SNR = 10 dB. It can be observed that doubling the number of RF antennas promotes the sum rate by approximately 3 bps/Hz. Increasing the number of phase candidates can also improve the performance, however, when *N*_BS_ equals 16 and 32, the advance is not significant. With more than 64 antennas, increasing the PSs will remarkably improve the channel capability. The system with *L* = 12 and *N*_BS_ = 128 increases the performance by about 3 bps/Hz than only two candidates, almost the same performance of doubling the number of RF antennas.

### 5.2. Performance of Proposed Hybrid-BD

In this subsection, we compare the achievable sum-rate performance of the proposed hybrid-BD, iterative optimization (IO) [36] which is incorporated in the proposed DSN, and equivalent hybrid BD whose spectral efficiency is defined as 1/*M* of the FC structure [41]. IO is developed to maximize the overall spectral efficiency by iteratively optimizing the contribution of each element in the RF processing matrix. The advance of the proposed algorithm lies in the fact that it is a global random search procedure, while IO may fall into the local optimal solution. The parameters of the proposed algorithm are set as follows: *S* = 500, *E* = 0.15*S*, and the maximum number of iterations of IO and the proposed algorithm are 10 and 45, respectively. Because the MSs are fully digital, the complete array gain is provided by the RF precoder at the BS.

**Figure 5:**
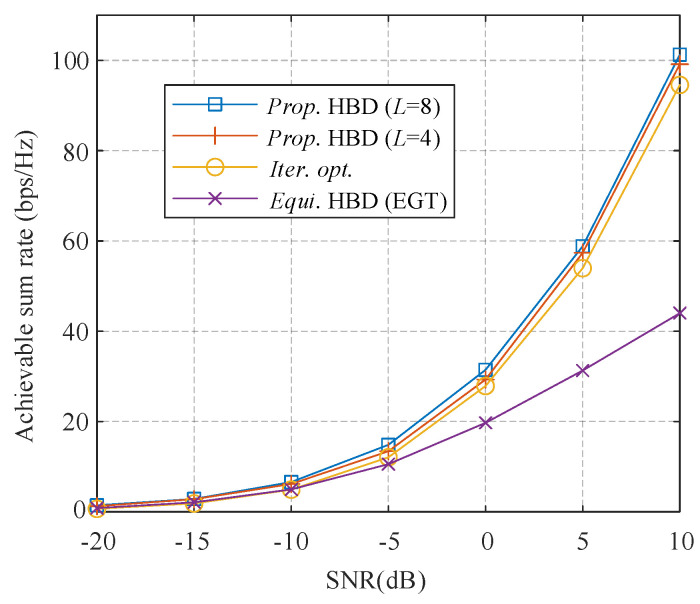
Achievable sum rate of different algorithms.

In Figure 5, we show the comparison of the achievable sum-rate where $M_{\mathrm{BS}}$ = 32 RF chains are employed to support *K* = 8 MSs, and each MS is scheduled with *N*s = 4 data streams. The antenna settings are 128 × 4, so each RF chain is connected to 4 antennas at the BS. It can be seen from the figure that the equivalent HBD with EGT is far from optimal, especially at a high SNR. The sum rate of the proposed scheme with 4 phase candidates surpasses the IO algorithm by about 0.5 dB and another 4 phase candidates can further promote about 0.3 dB over a wide range of SNR.

**Figure 6:**
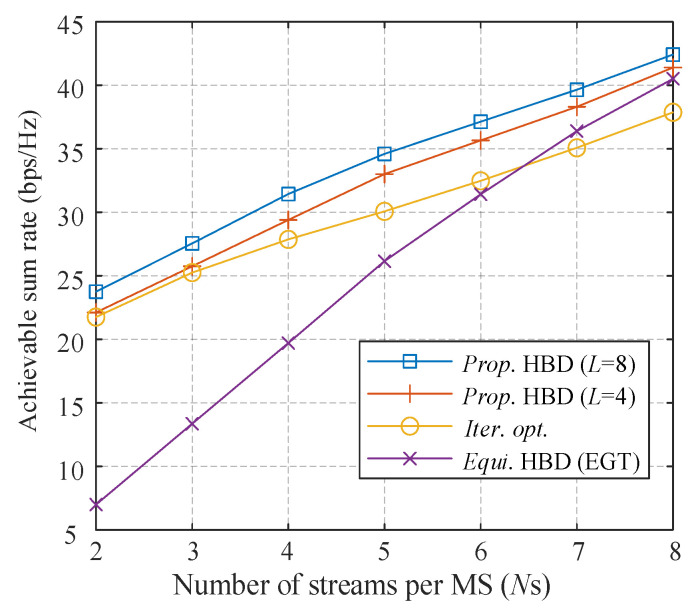
Achievable sum rate of different algorithms against the number of *N*s.

Figure 6 gives the spectral efficiency performance when the number of streams per MS varies from 2 to 8. The antenna configuration is 128 at the BS, and a totally *K* = 8 MSs are active simultaneously. The SNR is set to 0 dB. In all the schemes, we observe that when *N*s increases, the sum spectral efficiency grows almost linearly, and the proposed algorithm increases the performance by approximately 3 bps/Hz per stream. We can see that using EGT as the precoder fails to achieve the complete array gain, especially when few streams are adopted. From the figure, we can observe that the proposed algorithm with 8 phase candidates promotes about 2 bps/Hz compared with only 4 phase candidates.

**Figure 7:**
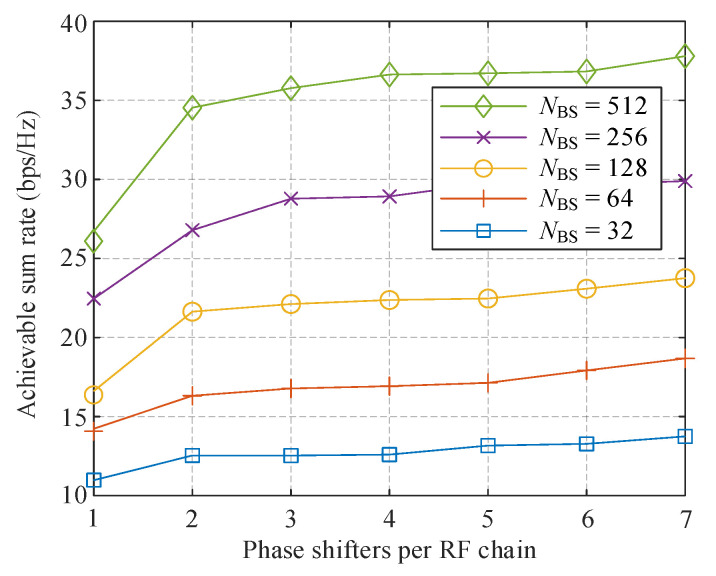
Comparison of achievable sum-rate with different *N*_BS_ against the number of PSs per RF chain.

In Figure 7, we compare the performance of the proposed algorithm with different RF antennas as a function of the number of phase candidates per RF chain. Totally *K* = 8 MSs and *N*s = 2 data streams per MS are supported simultaneously. The SNR is set to 0 dB. An Analog precoder with only one PS per RF chain is equivalent to the SI-based structure. It can be observed that doubling the number of RF antennas promotes the sum rate by about 5 bps/Hz. Increasing the number of phase candidates can also improve the performance, however, when $N_{\mathrm{BS}}$ equals 32 and 64, the benefit is not significant. With more than 128 antennas, multiple PSs per RF chain remarkably promotes the channel capacity than SI-based structure. A system with *L* = 8, $N_{\mathrm{BS}}$ = 128 advances two candidates’ case by about 7 bps/Hz, and comparable improvement can also be found when $N_{\mathrm{BS}}$ = 256 and 512.

### 5.3. Performance of Proposed Energy Efficiency Optimization Algorithm

**Figure 8:**
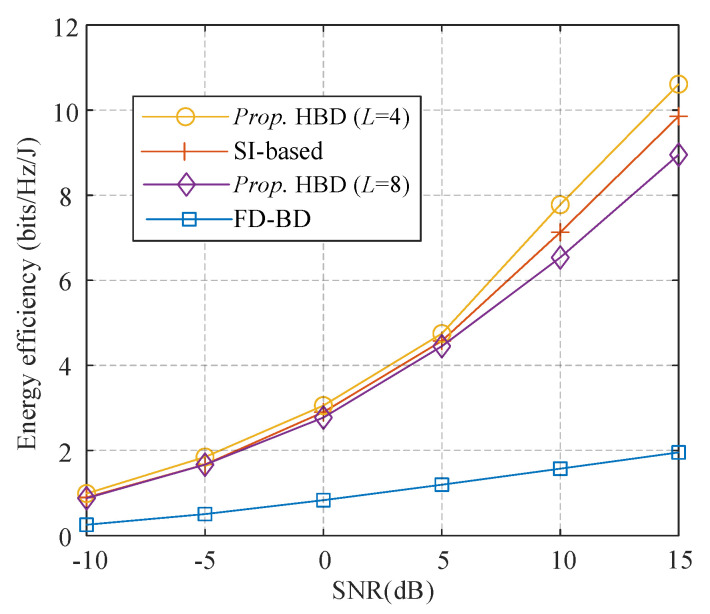
Energy efficiency comparison of different algorithms.

**Figure 9:**
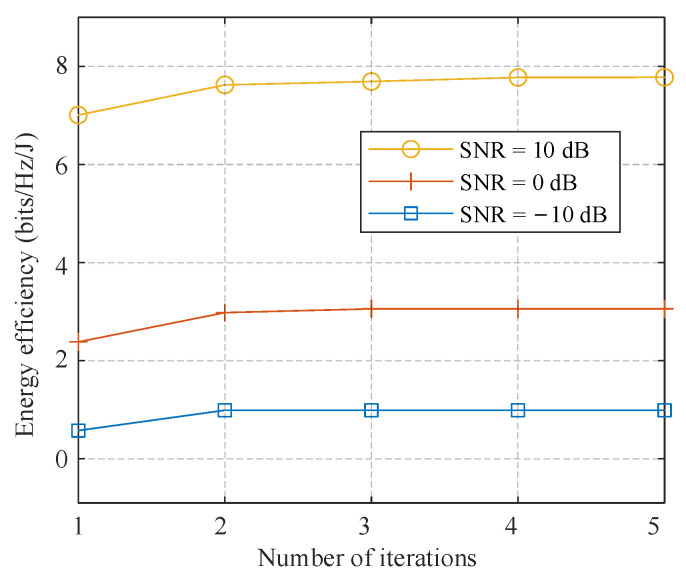
Convergence performance of the proposed algorithm with different SNR levels.

In this subsection, we evaluate the energy efficiency performance of the proposed algorithm where *N*_BS_ = 128 antennas, *K* = 8 users with *N*s = 2 streams are employed. Figure 8 shows the energy efficiency of the proposed algorithm with *L* = 2 (SI-based), 4, and 8, and fully-digital BD. It can be observed that hybrid precoding performs relatively better than fully digital solutions. The proposed algorithm with *L* = 4 outperforms those with *L* = 2 and 8 because it promotes the SE a lot at the cost of only 2 additional PSs per RF chain than the SI-based structure. For SNR at 10 dB, a proposed algorithm with *L* = 4 has an advantage of 0.7 bits/Hz/J than the SI-based structure, and about 6 bits/Hz/J better than the fully-digital solutions. Figure 9 shows the convergence of the algorithm proposed in Algorithm 3 to obtain the optimal number of active antennas. It can be observed that the energy efficiency of different SNR levels increases as the algorithm evolves. It converges rapidly and requires only 3 iterations to achieve an acceptable performance.

## 6. Conclusions

In this paper, a novel hybrid precoding structure based on DSN has been proposed for mmWave MU-MIMO downlink transmission. This structure uses fixed-degree PSs and switch networks rather than variable-degree PSs and RF adders to construct the analog precoder, which significantly reduces the hardware complexity and energy consumption. Hybrid-ZF and hybrid-BD are designed to cope with single-antenna and multiple-antenna MS scenarios, respectively. To obtain the RF precoding matrix, an evolving algorithm is proposed to optimize the distribution by minimizing the CE, and we formulate the closed-form solution to update the probability matrix in each iteration. We also show how to optimize the active number of antennas through DM-based fractional programming to maximize energy efficiency. The algorithm is updated dynamically based on the previously developed hybrid-ZF or BD. The performance of the proposed algorithms is examined with different configurations. Simulation results indicate that proposed schemes can match the channel to improve the users’ spectral efficiency and energy efficiency. Further study will be explored to minimize the population size and achieve faster convergency in future work.
